# Eye Contact Judgment Is Influenced by Perceivers’ Social Anxiety But Not by Their Affective State

**DOI:** 10.3389/fpsyg.2017.00373

**Published:** 2017-03-10

**Authors:** Tingji Chen, Lauri Nummenmaa, Jari K. Hietanen

**Affiliations:** ^1^Human Information Processing Laboratory, Faculty of Social Sciences/Psychology, University of TampereTampere, Finland; ^2^Turku PET Centre and Department of Psychology, University of TurkuTurku, Finland

**Keywords:** eye contact, affective state, olfaction, gaze cone, social anxiety

## Abstract

Fast and accurate judgment of whether another person is making eye contact or not is crucial for our social interaction. As affective states have been shown to influence social perceptions and judgments, we investigated the influence of observers’ own affective states and trait anxiety on their eye contact judgments. In two experiments, participants were required to judge whether animated faces (Experiment 1) and real faces (Experiment 2) with varying gaze angles were looking at them or not. Participants performed the task in pleasant, neutral, and unpleasant odor conditions. The results from two experiments showed that eye contact judgments were not modulated by observers’ affective state, yet participants with higher levels of social anxiety accepted a wider range of gaze deviations from the direct gaze as eye contact. We conclude that gaze direction judgments depend on individual differences in affective predispositions, yet they are not amenable to situational affective influences.

## Introduction

Fast and accurate discrimination of where another person is looking at, especially the judgment of whether another individual is making eye contact or not, is an important skill supporting social interaction. Not only humans use their eyes to capture visual information, but also to signal their social intentions (for a review, see Kleinke, 1986), and specialized neural systems subserve visual and social aspects of gaze processing (Nummenmaa and Calder, 2009).

Emotions influence how people think about and understand others and themselves in social settings (Forgas, 2000). Face perception and particularly perception of facial expressions is modulated by concurrent affective information emanating, for example, from the sender themselves as well as from the contextual information related to the physical environment and other surrounding people (for a review, see Wieser and Brosch, 2012). Perceivers’ own emotions also influence their perception of others’ facial expressions. Negative emotional state facilitates recognition of negative facial expressions and positive emotional state facilitates recognition of positive facial expressions (Schiffenbauer, 1974; Terwot et al., 1991; Bouhuys et al., 1995; Niedenthal et al., 2000; Leppänen and Hietanen, 2003; Forgas and East, 2008; Zhou and Chen, 2009; Schmid and Mast, 2010). Altogether these findings indicate that recognition of facial expressions is facilitated by affectively congruent contexts and perceivers’ emotions.

As eyes are the most salient feature in the face and gaze direction is a rich source of socially relevant information, a surprisingly limited number of studies have investigated the impact of emotion on gaze perception. Some previous studies have investigated gaze direction and eye contact perception in the context of emotional facial expressions. These studies have reported that averted gaze is identified faster when the face is fearful rather than angry, while direct gaze is identified faster when the face is angry rather than fearful (Adams and Franklin, 2009). Lobmaier et al. (2008, 2013) and Lobmaier and Perrett (2011) conducted a series of studies investigating the effects of facial expressions on perceived gaze direction. In these studies, they presented face pictures with different facial expressions and gaze angles to participants. Participants were required to judge whether the face was looking at them or not, or to indicate the perceived gaze direction by moving a slider. The results indicated that participants were more likely to interpret happy faces as looking at them as compared to angry, fearful, or neutral faces. Taken together, these studies suggested that gaze direction perception was modulated by contextual affective information provided by the gaze sender’s facial expression.

However, it remains unresolved whether gaze perception would be influenced by an observer’s *own* affective state, and the present study was designed to answer this question. As a communicative social signal, gaze direction both signals a sender’s approach-avoidance tendencies and activates corresponding motivational tendencies in the observer, thus, strongly regulating social connectedness (Argyle and Cook, 1976; Adams and Kleck, 2003, 2005; Hietanen et al., 2008; Wirth et al., 2010). Positive affect is associated with approach behavior and, thus, enhances a tendency of being cooperative and socializing, while a negative affect is associated with avoidance and leads to the opposite (Isen, 1987; Davidson et al., 1990; Davidson, 1996). Therefore, it could be expected that a person in a positive affective state would seek and perceive more social communication signals, i.e., more eye contact, as compared to a person in neutral and negative affective states.

To the best of our knowledge, there are no previous studies investigating the perceiver’s affective state on gaze perception. However, some affect-related traits, such as social anxiety, have been demonstrated to modulate individuals’ gaze perception. Individuals with social anxiety are prone to overestimate direct gaze from others (Gamer et al., 2011; Schulze et al., 2013; Bolt et al., 2014). Additionally, there are some studies investigating the influence of perceivers’ affect on their own gaze behavior. One study demonstrated that compared with the controls, individuals with induced positive affect established eye contact more often, whereas participants with induced negative affect had less frequent and shorter periods of eye contact with a confederate (Natale, 1977). Similar findings were reported in studies with clinically diagnosed patients. Depressed patients have been found to maintain shorter periods of eye contact and show more gaze aversion compared to control participants (Hinchliffe et al., 1970; Waxer, 1974).

In two experiments of the present study, we investigated the effects of a perceiver’s affective state on eye contact judgments. Additionally, we measured participants’ social anxiety in Experiment 2 to assess the potential interplay between affective state and trait variables. Pleasant, neutral, and unpleasant odors were used to induce corresponding affective states. Previous studies have demonstrated that olfactory stimuli are effective in influencing mood, reflected on both physiological and self-reported measures (Campenni et al., 2004; Herz, 2009; Porcherot et al., 2010). Importantly, manipulating affect by odors is unobtrusive and can be done simultaneously when participants are performing different tasks. Thus it is well suited for laboratory studies on emotion–cognition interactions. Odor-induced affects also modulate individuals’ cognitive processes and behavior (for a review, see Herz, 2002). Recognition of facial expressions has been shown to be facilitated when the odor context is affectively congruent with the expressions (Leppänen and Hietanen, 2003; Leleu et al., 2015), and odors can also influence the likability ratings of neutral faces (Li et al., 2007). Furthermore, studies have reported effects of odors on people’s approach-avoidance tendencies (for a brief review, see Holland et al., 2005; Zemke and Shoemaker, 2007). For example, a study on consumer behavior indicated that inoffensive scents (e.g., certain floral scents) compared to no scent in the environment, increased customers’ intentions to visit the store (Spangenberg et al., 1996). Pleasant ambient scents have also been shown to increase social interaction behaviors, e.g., eye contact, physical contact, and conversation, compared to a no scent condition (Zemke and Shoemaker, 2007). Thus, this evidence suggested that odors are well suited affect-inducers for the purpose of the present study.

## Experiment 1

In Experiment 1, participants viewed computer-generated faces with varying gaze angles and were required to judge whether the face was looking at them or not. Participants’ affective state was manipulated with pleasant, neutral, and unpleasant odors. To investigate the effect of odor-induced affects on eye contact judgments, we analyzed the width of gaze cone. Gaze cone refers to a width of gaze direction range perceived as eye contact and it has been used as a dependent variable in many previous studies investigating eye contact perception (e.g., Gamer and Hecht, 2007; Ewbank et al., 2009; Gamer et al., 2011; Uono and Hietanen, 2015; Lyyra et al., 2016). We expected that participants induced to have a positive affective state, as compared to neutral and negative affective states, would be prone to perceive a wider range of gaze directions as eye contact, i.e., would have wider width of gaze cone.

### Materials and Methods

#### Participants

Twenty-four participants (19 females, age range 19–34 years, mean 23 years) with self-reported normal or corrected-to-normal vision, normal sense of smell, and without any neurological or psychiatric diagnosis were recruited. All participants were informed about the general procedure of the experiment and they signed a consent form. They were requested not to wear any perfume or other products with strong smell. After the experiment, participants were given a movie ticket for their participation. The research protocol was approved by the Ethics Committee of the Tampere region.

#### Stimuli

**FIGURE 1:**
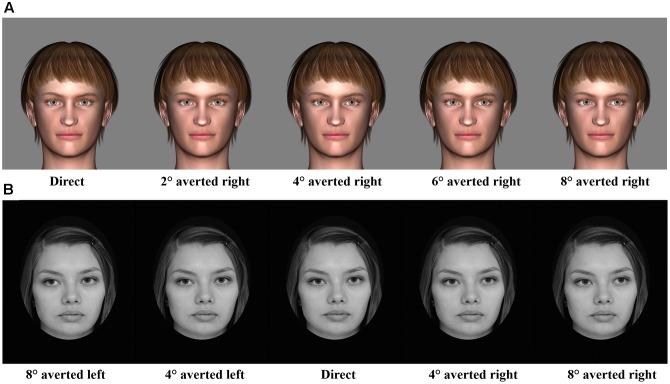
**Examples of gaze direction stimuli in Experiment 1** **(A)** and Experiment 2 **(B)**.

For odor stimuli, pyridine (Merck, 0.1% dilution), lemon essential oil (1% dilution), and water were used as an unpleasant, pleasant, and neutral odor, respectively. Pyridine and lemon essential oil have been effectively used as unpleasant and pleasant odors in prior studies (Leppänen and Hietanen, 2003). For gaze direction stimuli, eight characters (four males and four females) with nine different gaze angles (direct gaze and gaze averted 2, 4, 6, and 8° toward left and right) were created using a 3D animation software [Digital Art Zone (Daz) 3D Studio^1^] (**Figure 1A**). To avoid potential influence of facial asymmetry, all original stimuli were also presented horizontally flipped.

#### Procedure

The experiment was run using a fully within-subjects design. It consisted of three odor conditions, and the trials in each condition were presented in two blocks. The procedure in each condition was identical except that the odor was either unpleasant, pleasant, or neutral. The order of the odor conditions was counterbalanced between the participants. Before each condition, the experimenter prepared the odor apparatus. A container with a clean cotton swab inside was attached on a chinrest so that the swab was 7–8 cm away from the participant’s nose. Two milliliter of odor solution was dropped on the cotton at the beginning of each odor condition and 1 ml of liquid was added on the cotton between the blocks in order to keep the level of smell constant. A small air pump was connected to the bottom of the container to blow air into the container continuously during the experiment. Participants wore earmuffs to block low-frequency noise coming from the air pump. After each odor condition, participants took a 5-min break outside the testing room. The experimenter opened the windows until there was no detectable odor left in the room and then prepared the next odor condition. The air conditioning was always on in the testing room.

Each condition consisted of 80 trials with an equal number of 0, 2, 4, 6, and 8° averted gaze (half left/half right) trials. On each trial, a fixation cross was presented first in the center of the screen for 800 ms. This was followed by the presentation of the gaze stimulus for 150 ms. After the stimulus disappeared, participants were asked to make two judgments: first they judged whether the face was looking at them or not by pressing “1” or “2,” and immediately after this they evaluated the strength of their feeling of whether the face made eye contact or not on a 3-point scale (strong, intermediate, and weak) by pressing “1,” “2,” or “3.” The task was not paced, but response time (RT) was limited to 7 s. Participants gave responses with the numeric keypad on the right side of the keyboard. Response key mapping for the ‘looks at me’ task was counterbalanced across participants. After the participant’s second response, there was a 500-ms interval before the next trial. After each condition, participants rated the pleasantness of the odor using a scale ranging from 1 (unpleasant) to 9 (pleasant) presented on a computer screen.

#### Data Analysis

A one-way analysis of variance (ANOVA) was conducted on the odor ratings. For the eye contact judgment, five participants were excluded from the analysis because the manipulation check showed no differences in pleasantness ratings between odors for these participants. For computing the width of gaze cone, the proportion of looking-at-me responses was first calculated for each gaze angle separately in each odor condition. By using a binary logistic regression model for the proportion of looking-at-me responses data, the point at which a gaze stimulus had equal probabilities to be subjectively judged as eye contact or gaze aversion was calculated. This angle can be interpreted as the width of gaze deviation angle that an individual accepts as eye contact, i.e., gaze cone (Ewbank et al., 2009; Uono and Hietanen, 2015). Even though previous studies have suggested a symmetrical horizontal gaze cone (Vida and Maurer, 2012), it is still possible that left and right gaze cones are asymmetrically influenced by affective state. Thus, the width of gaze cone was calculated separately for gaze averted to the left and right. Because of the positive skew of the distribution of the angles, the gaze cone data were first normalized with a log10 transformation and then entered into a repeated-measures ANOVA with odor (pleasant, neutral, and unpleasant) and gaze direction (left and right) as within-subject factors. Responses to looking at me responses and the eye contact strength data were combined to range from 1 to 6 (1 = not looking at me, strong impression; 2 = not looking at me, intermediate impression; 3 = not looking at me, weak impression; 4 = looking at me, weak impression; 5 = looking at me, intermediate impression; 6 = looking at me, strong impression) and analyzed with a 3 (odor) × 5 (gaze angle) × 2 (gaze direction) ANOVA.

For violations of sphericity, a Huynh-Feldt correction procedure was applied. Least significant difference (LSD) test was performed for all multiple comparisons. For the sake of brevity, uncorrected degrees of freedom were reported. All statistical analyses were performed using the SPSS package.

### Result and Discussion

For the subjective pleasantness ratings, a one-way ANOVA showed a significant effect of odor, *F*(2,46) = 72.22, *p* < 0.001, $\eta_{p}^{2}$ = 0.758. Pairwise comparisons revealed that the smell of lemon (*M* = 7.08, *SE* = 0.27) was rated as significantly more pleasant than water (*M* = 4.75, *SE* = 0.16, *p* < 0.001) and water, in turn, was rated more pleasant than pyridine (*M* = 2.92, *SE* = 0.26, *p* < 0.001).

**FIGURE 2:**
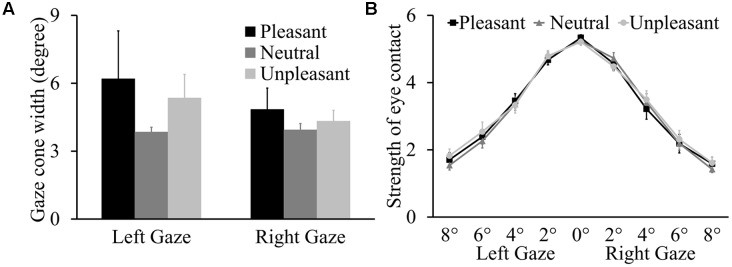
**(A)** Mean left and right gaze cone width with standard errors (SEs). **(B)** Mean strength of eye contact (±SE) for nine gaze angles as a function of three odor conditions.

The ANOVA on gaze cone width showed that the main effect of odor condition was not significant, *F*(2,36) = 0.76, *p* = 0.473, $\eta_{p}^{2}$ = 0.041, and there was no main effect of gaze direction (*p* = 0.339) or interaction between odor condition and gaze direction (*p* = 0.281) either (see **Figure 2A**). The proportions of looking-at-me responses for the nine gaze angles as a function of odor condition are presented in the Supplementary Table S1. For the eye contact strength rating data (**Figure 2B**), the analysis expectedly showed a main effect of gaze angle, *F*(4,72) = 188.50, *p* < 0.001, $\eta_{p}^{2}$ = 0.913. The strength of the eye contact feeling decreased with larger deviations of gaze angle from the direct gaze. Again, there was no main effect of odor condition or gaze direction (*p* = 0.754 and *p* = 0.284, respectively) or interactions involving odor condition, gaze angle, and gaze direction (*p* = 0.551, *p* = 0.640, *p* = 0.689, and *p* = 0.611).

Experiment 1 showed no effect of affective state on eye contact judgments. This was true both for the analyses based on the width of gaze cone and on the eye-contact strength ratings. The odor ratings indicated that our odor manipulation was, however, successful and a large number of previous studies have demonstrated the effect of odors on emotion, cognition, and behavior (for a review, see Herz, 2002). Particularly, a study using an identical odor manipulation procedure with the present experiment showed that participants recognized happy faces faster than faces expressing disgust in a pleasant odor context, whereas a reversed pattern of results was observed in an unpleasant odor context (Leppänen and Hietanen, 2003). One possibility for the lack of the odor effect could be that, although the gaze stimuli were briefly presented (150 ms), the participants were nevertheless given quite a long time to give their responses after the stimuli (7 s). Thus, the participants had ample time to evaluate the looking direction of the presented gaze stimulus and it is possible that this diminished the effect of odor context on eye contact judgments. To deal with this problem, in Experiment 2, we measured RTs and asked participants to respond as fast as possible.

It is also possible that the present results were affected by a confounding variable, i.e., by participants’ level of social anxiousness. Gamer et al. (2011) investigated gaze cone width in clinical samples with social anxiety. The results indicated that, compared to controls, participants with social anxiety showed a wider gaze cone, i.e., they were more likely to perceive averted gazes as being directed at them (Gamer et al., 2011). Through a web-based approach, another study showed a positive relationship between social anxiety and the direct gaze judgment: individuals with higher social anxiety scores had a stronger feeling to be looked at by others (Schulze et al., 2013). There is even evidence that, in participants with social anxiety disorder, the reduction of social anxiety symptoms as a result of Cognitive Behavioral Therapy is accompanied by decrease of the width of gaze cone (Harbort et al., 2013). Thus, social anxiety may have played an important, modulatory role in gaze perception in Experiment 1. Therefore, we took social anxiety into account in Experiment 2.

## Experiment 2

In Experiment 2, participants were required to judge five different gaze directions as either looking-at-me or not looking-at-me as fast as possible. Like in Experiment 1, the task was performed in three different affective contexts. We expected that such a setting would pressure participants to exhibit less controlled responses and, therefore, the task would be more sensitive to the influence of the odor context. Additionally, real faces instead of animated faces were used in Experiment 2 to increase realism of the stimuli. We also included the Social Phobia Scale (SPS, Mattick and Clarke, 1998) to control the potential influence of social anxiety.

### Materials and Methods

#### Participants

The recruitment procedures and inclusion criteria of participants were identical with those in Experiment 1. Twenty-eight participants (22 females, age range 20–39 years, mean 25 years) were enrolled in the present experiment.

#### Stimuli

Considering that, in Experiment 1, 5 participants (out of 24) did not show differences in their pleasantness ratings between odors, we increased the intensity of unpleasant odor (to 0.6% dilution) and replaced lemon with orange essential oil (1% dilution). Like in Experiment 1, water was used as a neutral odor. For gaze direction stimuli, grayscale photographic images of six Finnish persons (three males and three females) with five different gaze angles (direct gaze, 4 and 8° averted toward left and right) were selected from a set of stimuli prepared for a study by Uono and Hietanen (2015) (**Figure 1B**). All original stimuli were also presented horizontally flipped to avoid any potential influence of facial asymmetry.

#### Procedure

The procedures regarding the odor manipulation and block order were identical with those in Experiment 1. In each odor condition, the trials were presented in two blocks. Each block consisted of 48 trials with an equal number of direct, 4 and 8° averted gaze faces. On each experimental trial, first a fixation cross was presented in the center of the screen for 500 ms. This was followed by the presentation of the gaze stimulus for 500 ms. Participants were required to respond whether they felt that the person was “looking at me” or not as fast and accurate as possible by pressing “D” or “K” on the keyboard. They were allowed to give their response within a time-window of 3500 ms starting from the stimulus onset. Response key location was counterbalanced across participants. The inter-trial interval was 1000 ms. After each condition, participants were required to rate the pleasantness of the odor from 1 to 9 (1 = unpleasant, 9 = pleasant).

After the computer task, all participants completed the Social Phobia Scale (SPS) which consists of 20 items and each item is rated on a five-point Likert scale ranging from 0 (not at all) to 4 (extremely) (Mattick and Clarke, 1998).

#### Data Analysis

A one-way ANOVA was conducted on the odor ratings. For the eye contact judgment, four participants were excluded from the analysis because the manipulation check showed no differences in pleasantness ratings between odors for these participants. Two more participants were excluded due to low response accuracy for direct and 8° averted gaze (69 and 64%, respectively). For each participant, we calculated both the gaze cone width and the RT for each gaze angle (separately for the gaze averted to the left and right) in each odor condition. A repeated-measures ANOVA with odor condition and gaze direction as within-subject factors was performed on the gaze cone data. For the RT data, the data from trials with incorrect responses to the gaze directions (16%) and trials with response latencies shorter than 2.5 standard deviations (SDs) below or longer than 2.5 SDs above each participant’s mean (1.7%) were excluded. The averaged RT data were first normalized with a log10 transformation and then entered into a 3 (odor condition: pleasant, neutral, and unpleasant) × 3 (gaze angle: 0, 4, and 8°) × 2 (gaze direction: left, right) ANOVA.

The possible modulating influence of social anxiety on gaze cone width in different odor conditions was investigated using an analysis of covariance (ANCOVA) with odor condition and gaze direction as repeated-measures factors and SPS score as a covariate.

### Results and Discussion

For the pleasantness ratings of odors, there was a significant effect of odor, *F*(2,54) = 65.62, *p* < 0.001, $\eta_{p}^{2}$ = 0.709. Pairwise comparisons revealed that the smell of orange (*M* = 6.07, *SE* = 0.35) was rated as significantly more pleasant than water (*M* = 4.71, *SE* = 0.18, *p* = 0.001) and water, in turn, was rated more pleasant than pyridine (*M* = 2.04, *SE* = 0.21, *p* < 0.001).

**FIGURE 3:**
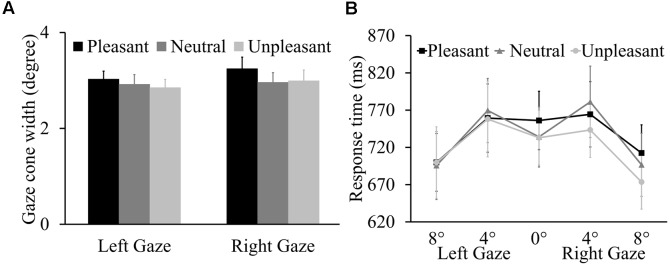
**(A)** Mean left and right gaze cone width with SEs. **(B)** Mean response times (RTs) (±SE) for five gaze angles as a function of three odor conditions.

The ANOVA on gaze cone width showed no main effect of odor condition, *F*(2,42) = 0.78, *p* = 0.467, $\eta_{p}^{2}$ = 0.036. Additionally, there was no main effect of gaze direction (*p* = 0.389) or interaction between odor condition and gaze direction (*p* = 0.773) (**Figure 3A**). The proportions of looking-at-me responses for the five gaze angles as a function of odor condition are presented in the Supplementary Table S2. For the RT data (**Figure 3B**), there was a main effect of gaze angle, *F*(2,42) = 20.03, *p* < 0.001, $\eta_{p}^{2}$ = 0.488. Overall, participants were faster to respond to 8° averted gaze (*M* = 696 ms, *SE* = 38.21) than to direct gaze (*M* = 741 ms, *SE* = 36.63, *p* = 0.006) and to 4° averted gaze (*M* = 762 ms, *SE* = 41.32, *p* < 0.001). Importantly, there was no main effect of odor condition or gaze direction (*p* = 0.639 and *p* = 0.845, respectively) or interactions involving odor condition, gaze angle, and gaze direction (*p* = 0.168, *p* = 0.283, *p* = 0.964, and *p* = 0.596).

**FIGURE 4:**
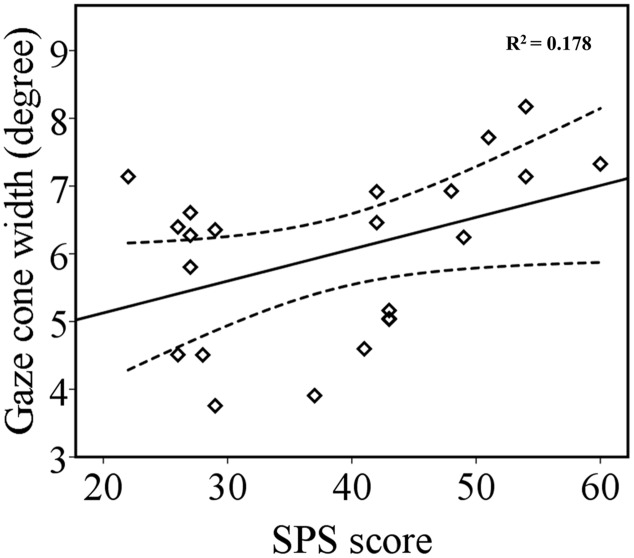
**The relationship between gaze cone width and SPS scores.** The regression line (solid line) and upper and lower 95% CIs (dashed line) are shown.

The ANCOVA on gaze cone width showed that there was no main effect of odor condition or gaze direction (*p* = 0.797 and *p* = 0.333, respectively) or interaction involving odor condition, gaze direction, and social anxiety score (*p* = 0.291, *p* = 0.756, *p* = 0.213, and *p* = 0.303). Importantly, the results revealed a significant main effect of social anxiety, *F*(1,20) = 4.34, *p* = 0.050, $\eta_{p}^{2}$ = 0.178. A correlation analysis between each participant’s gaze cone width (left and right gaze cone combined and averaged across odor conditions) and SPS scores showed a positive correlation between gaze cone width and SPS scores (*r* = 0.422, *p* = 0.025, one-tailed) (**Figure 4**). Thus, participants with higher levels of social anxiety tended to have a wider gaze cone, that is to say, they were more likely to perceive a gaze as being directed at them. The correlation between each participant’s average RT and SPS score was not significant (*r* = 0.287, *p* = 0.196).

Finally, in order to increase the power of statistical testing, we pooled the left and right gaze cone width data from Experiments 1 and 2 and performed a repeated-measures ANOVA. Again, the results showed no main effect of odor condition (*p* = 0.235), main effect of gaze direction (*p* = 0.494) or interaction between odor condition and gaze direction (*p* = 0.458). From the pooled data, we also analyzed the 95% confidence intervals (CIs) for the differences between the odor conditions. These were: -0.7 ≤ CI (μ_pleasant_ - μ_neutral_) ≤ 2.5; -0.8 ≤ CI (μ_pleasant_ - μ_unpleasant_) ≤ 1.7; -1.2 ≤ CI (μ_neutral_ - μ_unpleasant_) ≤ 0.4. These CIs are rather small indicating that with a high probability there were no differences in gaze cone width between different odor conditions.

In Experiment 2, we analyzed both gaze cone width and the RTs to eye contact judgment in pleasant, neutral, and unpleasant odor conditions. The results replicated the findings of Experiment 1: eye contact judgments were not modulated by perceivers’ affective state, even after controlling for social anxiety scores. In addition, the present experiment replicated the results of previous studies showing that participants with higher levels of social anxiety interpreted wider gaze deviations from a true direct gaze as an eye contact (Gamer et al., 2011; Schulze et al., 2013).

## General Discussion

Our main finding was that across two experiments, neither positive nor negative affective state influenced eye contact judgment. Instead, individual differences in social anxiety were associated with eye contact judgments, with high anxiety leading to more liberal criterion in the gaze contact detection.

We start discussing these findings by asking first how affective states come to influence our social judgments. It has been suggested that individuals’ own affective states may be used as diagnostic information when making social judgments regarding other people, because it provides information about the elicited action tendencies toward others (Schwarz, 1990; Forgas, 1995; Gendolla, 2000). For example, in judging the likability of another person, individuals with a positive affect mistakenly interpret their own positive affect as their feeling about the judged person, and evaluate the target person more positively when in a happy rather than a sad mood (Gendolla, 2000; Schwarz, 2000). Another possibility is that an affective state may activate associated concepts, words, themes, and inference rules, and these activated representations will become more likely to be accessed in subsequent judgments (Bower et al., 1983). Consequently, “these mental sets then act as interpretive filters of reality” and bias individuals’ social judgments (Bower et al., 1983, p. 395). Via these mechanisms, affective states lead to affect-congruent judgments. Thus, affective states may influence, for example, predictions regarding the weather (sunny-rainy), one’s own future (optimistic-pessimistic), the likability ratings of a neutral face (likable–dislikable), and the recognition of facial expressions (happy–sad) (Johnson and Tversky, 1983; Bower, 1991; Mayer et al., 1992; Bouhuys et al., 1995; Li et al., 2007). However, if the judgment to-be-made as such is not strongly associated with a positive or negative affect, the affective state may not exert its influence on the judgment. Indeed, this may be the case in the present study regarding the eye contact judgment: Evaluating whether another person is looking at me or not is not an affective judgment as such. Therefore, the present results suggest that the affective congruency effects, whether based on affect as information type of explanations (Schwarz and Clore, 1983) or spreading activation theories (Bower, 1981), exert strong influences in instances where the social judgment as such is affect-related, but less so in situations where the social decision is devoid of affective contents.

The present results also suggest that, in contrast to a relatively robust congruency effects in affective judgments (Bower, 1981; Mayer et al., 1992), corresponding congruency effect is absent for approach-avoidance motivation. We hypothesized that by inducing positive (approach) and negative (avoidance) affective states in individuals, we could influence participants’ evaluations of others’ approach/avoidance tendencies inferred from their eye gaze. The present results, however, imply that approach and avoidance motivation does not sensitize an observer to perceive congruent social signals in their environment similarly as affects do. This accords with recent data showing that when recognizing morphed expressions of anger, an unpleasant (versus pleasant or neutral) odor significantly lowered the morphing threshold required for accurate recognition (Leleu et al., 2015). Thus, even if the unpleasant odor context had induced avoidance motivation in the participants, they, nevertheless, were more sensitive to valence-congruent, but motivation-incongruent facial expression of anger. Anger is considered to be an affectively negative emotion, but associated with an approach motivation (for a brief review, see Harmon-Jones and Sigelman, 2001).

Affect-related states, such as stress, social anxiety, and feelings of ostracism, increase the likelihood of interpreting a gaze as looking toward the self (Gamer et al., 2011; Rimmele and Lobmaier, 2012; Schulze et al., 2013; Lyyra et al., 2016). If affective states do not influence judgments of gaze direction, as postulated above, how then these studies have shown the effect? The reason likely relates to that although all these states involve (negative) affects, the affective state *per se* was not the cause for the biases in the gaze direction judgments. For example, stress occurs when individuals perceive that they do not have sufficient sources to cope with a threatening or a demanding situation (Cohen et al., 1983). In such situations, it is adaptive to become more alert and self-centered and, consequently, individuals are more likely to interpret another person’s gaze as directed toward themselves (Rimmele and Lobmaier, 2012). Social anxiety is characterized by an intense fear and avoidance of social situations in which an individual may be scrutinized by others (American Psychiatric Association, 2013) and, at the same time, individuals with social anxiety also show biases in information processing which drives them more likely to interpret social situations in a negative way (Clark and McManus, 2002). Indeed, compared to controls, individuals with social anxiety experience eye contact as aversive (Myllyneva et al., 2015), and are also prone to interpret a gaze direction as being directed at them (Gamer et al., 2011; Schulze et al., 2013). Being ostracized does not only evoke negative affect, but also lowers one’s experience of fulfillment of the basic needs (e.g., belongingness) (Wirth et al., 2010). Lyyra et al. (2016) suggested that socially excluded individuals are biased toward gaze contact detection due to their need for reaffiliation. Thus, even though previous research has shown increased eye contact perception caused by stress, social anxiety, and feelings of ostracism, these studies cannot be considered as having provided evidence that affective states as such would influence the gaze direction judgments.

Although the present results did not show the influence of affective state on eye-contact judgments, we found a statistically significant correlation between the width of gaze cone and self-reported social anxiety. In accordance with prior studies, participants with higher levels of social anxiety accepted a wider range of gaze directions as eye contact (Gamer et al., 2011; Jun et al., 2013; Schulze et al., 2013).

Frick (1995) proposed a good-effort criterion for accepting the null hypothesis. He suggested that once a good effort has been made to find the effect but none has been found, the null hypothesis should be accepted. An objective criterion for a good effort is demonstrating a related effect (Frick, 1995). In our study, we demonstrated this by finding a significant positive correlation between gaze cone width and social anxiety. This related effect indicated that the present study was methodologically sound, it had a sufficient number of participants and trials, and the variances were well-controlled. Furthermore, the CIs analyzed from the data pooled across Experiments 1 and 2 were small; small CIs have also been considered as one of the criteria of good effort to find an effect (Frick, 1995).

A limitation of the present study is that although we used a valence based classification in our odor manipulation conditions (positive, neutral, and negative), it is a fact that distinct emotions with the same valence differentially influence thoughts and judgments (Raghunathan and Pham, 1999; Lerner and Keltner, 2000). For example, people’s assessments of risk probabilities have been shown to be positively related to their level of dispositional fear but negatively related to their level of dispositional anger (Lerner and Keltner, 2000). The authors explained their findings by suggesting that each emotion activated a corresponding appraisal pattern. This appraisal pattern automatically guided subsequent perceptions and judgments. For example, fear activated an appraisal tendency to perceive negative events as uncertain and outside of personal control, and consequently led to pessimistic risk perception. On the contrary, anger activated an appraisal tendency to perceive negative events as certain and under personal control, consequently leading to optimistic perceptions (Smith and Ellsworth, 1985; Lerner and Keltner, 2000). In the present studies, we manipulated the affective context by using olfactory stimuli of lemon/orange, water, and pyridine. The smell of pyridine was unpleasant and negative to the participants, as shown by their self-ratings, and, for most people, the smell was specifically disgusting. Thus, it is likely that it automatically activated a different appraisal tendency as compared to other emotions with negative valence, e.g., sadness, anger, and fear. Future studies should investigate the eye contact perception with a broader variety of induced emotions.

## Conclusion

We conclude that observers’ affective state does not influence their eye contact judgments. The results were consistent when considering both the width of gaze cone and RTs, and across two experiments using both animated and real face pictures as stimuli. Consistent with previous studies, our study, however, showed a positive relationship between gaze cone width and social anxiety. We suggest that affective states are not likely to influence social judgments if the judgments *per se* are not related to evaluations involving the dimension of affective valence.

## Author Contributions

TC and JH were involved in the experimental design, data collection and analysis. All the three authors were involved in manuscript writing and data interpretation work.

## Conflict of Interest Statement

The authors declare that the research was conducted in the absence of any commercial or financial relationships that could be construed as a potential conflict of interest.

## Footnotes

1 http://www.daz3d.com

## References

[B1] Adams R. B. Jr. Franklin R. G. Jr. (2009). Influence of emotional expression on the processing of gaze direction. *Motiv. Emot.* 33 106–112. 10.1007/s11031-009-9121-9

[B2] Adams R. B. Kleck R. E. (2003). Perceived gaze direction and the processing of facial displays of emotion. *Psychol. Sci.* 14 644–647. 10.1046/j.0956-7976.2003.psci_1479.x 14629700

[B3] Adams R. B. Kleck R. E. (2005). Effects of direct and averted gaze on the perception of facially communicated emotion. *Emotion* 5 3–11. 10.1037/1528-3542.5.1.3 15755215

[B4] American Psychiatric Association (2013). *Diagnostic and Statistical Manual of Mental Disorders (DSM-5)*, 5th Edn Washington, DC: American Psychiatric Association.

[B5] Argyle M. Cook M. (1976). *Gaze and Mutual Gaze.* New York, NY: Cambridge University Press.

[B6] Bolt O. C. Ehlers A. Clark D. M. (2014). Faces in a crowd: high socially anxious individuals estimate that more people are looking at them than low socially anxious individuals. *PLoS ONE* 9:e106400 10.1371/journal.pone.0106400 PMC4160315 25208221

[B7] Bouhuys A. L. Bloem G. M. Groothuis T. G. G. (1995). Induction of depressed and elated mood by music influences the perception of facial emotional expressions in healthy-subjects. *J. Affect. Disord.* 33 215–226. 10.1016/0165-0327(94)00092-n 7790675

[B8] Bower G. H. (1981). Mood and memory. *Am. Psychol.* 36 129–148. 10.1037//0003-066x.36.2.129 7224324

[B9] Bower G. H. (1991). “Mood congruity of social judgments,” in *Emotion and Social Judgments*, ed. Forgas J. P. (Elmsford, NY: Pergamon Press), 31–53.

[B10] Bower G. H. Sahgal A. Routh D. (1983). Affect and cognition [and discussion]. *Philos. Trans. R. Soc. B Biol. Sci.* 302 387–402. 10.1098/rstb.1983.0062

[B11] Campenni C. E. Crawley E. J. Meier M. E. (2004). Role of suggestion in odor-induced mood change. *Psychol. Rep.* 94 1127–1136. 10.2466/pr0.94.3c.1127-1136 15362382

[B12] Clark D. M. McManus F. (2002). Information processing in social phobia. *Biol. Psychiatry* 51 92–100. 10.1016/s0006-3223(01)01296-3 11801234

[B13] Cohen S. Kamarck T. Mermelstein R. (1983). A global measure of perceived stress. *J. Health Soc. Behav.* 24 385–396. 10.2307/2136404 6668417

[B14] Davidson R. J. (1996). “Cerebral asymmetry, emotion, and affective style,” in *Brain Asymmetry*, eds Davidson R. J. Hugdahl K. (Cambridge, MA: Massachusetts Institute of Technology), 361–387.

[B15] Davidson R. J. Saron C. D. Senulis J. A. Ekman P. Friesen W. V. (1990). Approach withdrawal and cerebral asymmetry - emotional expression and brain physiology I. *J. Pers. Soc. Psychol.* 58 330–341. 10.1037/0022-3514.58.2.330 2319445

[B16] Ewbank M. P. Jennings C. Calder A. J. (2009). Why are you angry with me? Facial expressions of threat influence perception of gaze direction. *J. Vis.* 9 1–7. 10.1167/9.12.16 20053107

[B17] Forgas J. P. (1995). Mood and judgment: the affect infusion model (AIM). *Psychol. Bull.* 117 39–66. 10.1037/0033-2909.117.1.39 7870863

[B18] Forgas J. P. East R. (2008). How real is that smile? Mood effects on accepting or rejecting the veracity of emotional facial expressions. *J. Nonverbal Behav.* 32 157–170. 10.1007/s10919-008-0050-1

[B19] Forgas J. P. (ed.) (2000). *Feeling and Thinking: The Role of Affect in Social Cognition.* Cambridge: Cambridge University Press.

[B20] Frick R. W. (1995). Accepting the null hypothesis. *Mem. Cogn.* 23 132–138. 10.3758/bf03210562 7885262

[B21] Gamer M. Hecht H. (2007). Are you looking at me? Measuring the cone of gaze. *J. Exp. Psychol.Hum. Percept. Perform.* 33 705–715. 10.1037/0096-1523.33.3.705 17563231

[B22] Gamer M. Hecht H. Seipp N. Hiller W. (2011). Who is looking at me? The cone of gaze widens in social phobia. *Cogn. Emot.* 25 756–764. 10.1080/02699931.2010.503117 21547777

[B23] Gendolla G. H. (2000). On the impact of mood on behavior: an integrative theory and a review. *Rev. Gen. Psychol.* 4:378 10.1037/1089-2680.4.4.378

[B24] Harbort J. Witthoeft M. Spiegel J. Nick K. Hecht H. (2013). The widening of the gaze cone in patients with social anxiety disorder and its normalization after CBT. *Behav. Res. Ther.* 51 359–367. 10.1016/j.brat.2013.03.009 23639302

[B25] Harmon-Jones E. Sigelman J. (2001). State anger and prefrontal brain activity: evidence that insult-related relative left-prefrontal activation is associated with experienced anger and aggression. *J. Pers. Soc. Psychol.* 80 797–803. 10.1037/0022-3514.80.5.797 11374750

[B26] Herz R. S. (2002). “Influences of odors on mood and affective cognition,” in *Olfaction, Taste, and Cognition*, eds Rouby C. Schaal B. Dubois D. Gervais R. Holley A. (Cambridge: Cambridge University Press), 160–177.

[B27] Herz R. S. (2009). Aromatherapy facts and fictions: a scientific analysis of olfactory effects on mood, physiology and behavior. *Int. J. Neurosci.* 119 263–290. 10.1080/00207450802333953 19125379

[B28] Hietanen J. K. Leppänen J. M. Peltola M. J. Linna-aho K. Ruuhiala H. J. (2008). Seeing direct and averted gaze activates the approach-avoidance motivational brain systems. *Neuropsychologia* 46 2423–2430. 10.1016/j.neuropsychologia.2008.02.029 18402988

[B29] Hinchliffe M. Lancashire M. Roberts F. J. (1970). Eye-contact and depression: a preliminary report. *Br. J. Psychiatry* 117 571–572. 10.1192/bjp.117.540.571 5480708

[B30] Holland R. W. Hendriks M. Aarts H. (2005). Smells like clean spirit. *Psychol. Sci.* 16 689–693. 10.1111/j.1467-9280.2005.01597.x 16137254

[B31] Isen A. M. (1987). Positive affect, cognitive processes, and social behavior. *Adv. Exp. Soc. Psychol.* 20 203–253. 10.1016/s0065-2601(08)60415-3

[B32] Johnson E. J. Tversky A. (1983). Affect, generalization, and the perception of risk. *J. Pers. Soc. Psychol.* 45:20 10.1037/0022-3514.45.1.20

[B33] Jun Y. Y. Mareschal I. Clifford C. W. G. Dadds M. R. (2013). Cone of direct gaze as a marker of social anxiety in males. *Psychiatry Res.* 210 193–198. 10.1016/j.psychres.2013.05.020 23769393

[B34] Kleinke C. L. (1986). Gaze and eye contact: a research review. *Psychol. Bull.* 100 78–100. 10.1037//0033-2909.100.1.78 3526377

[B35] Leleu A. Demily C. Franck N. Durand K. Schaal B. Baudouin J.-Y. (2015). The odor context facilitates the perception of low-intensity facial expressions of emotion. *PLoS ONE* 10:e0138656 10.1371/journal.pone.0138656 PMC4577100 26390036

[B36] Leppänen J. M. Hietanen J. K. (2003). Affect and face perception: odors modulate the recognition advantage of happy faces. *Emotion* 3 315–326. 10.1037/1528-3542.3.4.315 14674826

[B37] Lerner J. S. Keltner D. (2000). Beyond valence: toward a model of emotion-specific influences on judgement and choice. *Cogn. Emot.* 14 473–493. 10.1080/026999300402763

[B38] Li W. Moallem I. Paller K. A. Gottfried J. A. (2007). Subliminal smells can guide social preferences. *Psychol. Sci.* 18 1044–1049. 10.1111/j.1467-9280.2007.02023.x 18031410

[B39] Lobmaier J. S. Hartmann M. Volz A. J. Mast F. W. (2013). Emotional expression affects the accuracy of gaze perception. *Motiv. Emot.* 37 194–201. 10.1007/s11031-012-9295-4

[B40] Lobmaier J. S. Perrett D. I. (2011). The world smiles at me: self-referential positivity bias when interpreting direction of attention. *Cogn. Emot.* 25 334–341. 10.1080/02699931003794557 21432675

[B41] Lobmaier J. S. Tiddeman B. P. Perrett D. I. (2008). Emotional expression modulates perceived gaze direction. *Emotion* 8 573–577. 10.1037/1528-3542.8.4.573 18729587

[B42] Lyyra P. Wirth J. H. Hietanen J. K. (2016). Are you looking my way? Ostracism widens the cone of gaze. *Q. J. Exp. Psychol.* 70 1713–1721. 10.1080/17470218.2016.1204327 27327894

[B43] Mattick R. P. Clarke J. C. (1998). Development and validation of measures of social phobia scrutiny fear and social interaction anxiety. *Behav. Res. Ther.* 36 455–470. 10.1016/s0005-7967(97)10031-6 9670605

[B44] Mayer J. D. Gaschke Y. N. Braverman D. L. Evans T. W. (1992). Mood-congruent judgment is a general effect. *J. Pers. Soc. Psychol.* 63 119–132. 10.1037//0022-3514.63.1.119

[B45] Myllyneva A. Ranta K. Hietanen J. K. (2015). Psychophysiological responses to eye contact in adolescents with social anxiety disorder. *Biol. Psychol.* 109 151–158. 10.1016/j.biopsycho.2015.05.005 26032869

[B46] Natale M. (1977). Induction of mood states and their effect on gaze behaviors. *J. Consult. Clin. Psychol.* 45:960 10.1037/0022-006x.45.5.960

[B47] Niedenthal P. M. Halberstadt J. B. Margolin J. Innes-Ker A. H. (2000). Emotional state and the detection of change in facial expression of emotion. *Eur. J. Soc. Psychol.* 30 211–222.

[B48] Nummenmaa L. Calder A. J. (2009). Neural mechanisms of social attention. *Trends Cogn. Sci.* 13 135–143. 10.1016/j.tics.2008.12.006 19223221

[B49] Porcherot C. Delplanque S. Raviot-Derrien S. Le Calve B. Chrea C. Gaudreau N. (2010). How do you feel when you smell this? Optimization of a verbal measurement of odor-elicited emotions. *Food Qual. Prefer.* 21 938–947. 10.1016/j.foodqual.2010.03.012

[B50] Raghunathan R. Pham M. T. (1999). All negative moods are not equal: motivational influences of anxiety and sadness on decision making. *Organ. Behav. Hum. Decis. Process.* 79 56–77. 10.1006/obhd.1999.2838 10388609

[B51] Rimmele U. Lobmaier J. S. (2012). Stress increases the feeling of being looked at. *Psychoneuroendocrinology* 37 292–298. 10.1016/j.psyneuen.2011.06.013 21767917

[B52] Schiffenbauer A. (1974). Effect of observer’s emotional state on judgments of the emotional state of others. *J. Pers. Soc. Psychol.* 30 31–35. 10.1037/h0036643 4427214

[B53] Schmid P. C. Mast M. S. (2010). Mood effects on emotion recognition. *Motiv. Emot.* 34 288–292. 10.1007/s11031-010-9170-0

[B54] Schulze L. Lobmaier J. S. Arnold M. Renneberg B. (2013). All eyes on me?! Social anxiety and self-directed perception of eye gaze. *Cogn. Emot.* 27 1305–1313. 10.1080/02699931.2013.773881 23438447

[B55] Schwarz N. (1990). “Feelings as information: informational and motivational functions of affective states,” in *Handbook of Motivation and Cognition: Foundations of Social Behavior*, eds Higgins E. T. Sorrentino R. M. (New York, NY: Guilford Press), 527–561.

[B56] Schwarz N. (2000). Emotion, cognition, and decision making. *Cogn. Emot.* 14 433–440. 10.1080/026999300402745

[B57] Schwarz N. Clore G. L. (1983). Mood, misattribution, and judgments of well-being: informative and directive functions of affective states. *J. Pers. Soc. Psychol.* 45:513 10.1037/0022-3514.45.3.513

[B58] Smith C. A. Ellsworth P. C. (1985). Patterns of cognitive appraisal in emotion. *J. Pers. Soc. Psychol.* 48 813–838. 10.1037//0022-3514.48.4.813 3886875

[B59] Spangenberg E. R. Crowley A. E. Henderson P. W. (1996). Improving the store environment: do olfactory cues affect evaluations and behaviors? *J. Mark.* 60 67–80. 10.2307/1251931

[B60] Terwot M. M. Kremera H. H. Steggea H. (1991). Effects of children’s emotional state on their reactions to emotional expressions: a search for congruency effects. *Cogn. Emot.* 5 109–121. 10.1080/02699939108411028

[B61] Uono S. Hietanen J. K. (2015). Eye contact perception in the west and east: a cross-cultural study. *PLoS ONE* 10:e0118094 10.1371/journal.pone.0118094 PMC4340785 25714900

[B62] Vida M. D. Maurer D. (2012). The development of fine-grained sensitivity to eye contact after 6years of age. *J. Exp. Child Psychol.* 112 243–256. 10.1016/j.jecp.2012.02.002 22417921

[B63] Waxer P. (1974). Nonverbal cues for depression. *J. Abnorm. Psychol.* 83 319–322. 10.1037/h0036706 4844922

[B64] Wieser M. J. Brosch T. (2012). Faces in context: a review and systematization of contextual influences on affective face processing. *Front. Psychol.* 3:471 10.3389/fpsyg.2012.00471 PMC3487423 23130011

[B65] Wirth J. H. Sacco D. F. Hugenberg K. Williams K. D. (2010). Eye gaze as relational evaluation: averted eye gaze leads to feelings of ostracism and relational devaluation. *Pers. Soc. Psychol. Bull.* 36 869–882. 10.1177/0146167210370032 20505162

[B66] Zemke D. M. V. Shoemaker S. (2007). Scent across a crowded room: exploring the effect of ambient scent on social interactions. *Int. J. Hosp. Manag.* 26 927–940. 10.1016/j.ijhm.2006.10.009

[B67] Zhou W. Chen D. (2009). Fear-related chemosignals modulate recognition of fear in ambiguous facial expressions. *Psychol. Sci.* 20 177–183. 10.1111/j.1467-9280.2009.02263.x 19170944

