# Supplementary material for: Eye Contact Judgment Is Influenced by Perceivers’ Social Anxiety But Not by Their Affective State
Source: Front Psychol. 2017 Mar 10;8:373. doi: 10.3389/fpsyg.2017.00373 (PMC5344928; doi:10.3389/fpsyg.2017.00373)
Supplement: Supplementary file 1 [file Table_1.PDF]

Table S1.

The proportion (%) of looking-at-me responses for nine gaze angles in three odor conditions. Left gaze (-), right gaze (+).

	-					+			
	8°	6°	4°	2°	0°	2°	4°	6°	8°
Pleasant	10.5	22.4	47.4	78.9	94.4	73.7	41.4	21.1	9.2
Neutral	3.9	19.1	42.1	80.9	90.1	78.9	49.3	19.1	4.6
Unpleasant	11.2	27.0	44.1	80.3	92.4	76.3	49.3	23.0	7.9
