# Supplementary material for: Eye Contact Judgment Is Influenced by Perceivers’ Social Anxiety But Not by Their Affective State
Source: Front Psychol. 2017 Mar 10;8:373. doi: 10.3389/fpsyg.2017.00373 (PMC5344928; doi:10.3389/fpsyg.2017.00373)
Supplement: Supplementary file 2 [file Table_2.PDF]

Table S2

The proportion (%) of looking-at-me responses for five gaze angles in three odor conditions. Left gaze (-), right gaze (+).

	-			+	
	8°	4°	0°	4°	8°
Pleasant	4.3	31.5	85.4	32.7	8.0
Neutral	3.4	28.1	85.8	29.0	3.7
Unpleasant	4.3	26.1	85.4	30.7	5.1
